# Accelerometer-Based Handheld Navigation Instrumentation in Total Knee Arthroplasty Decrease Blood Loss Compared to Conventional Instrumentation: A Prospective Comparative Study

**DOI:** 10.7759/cureus.32589

**Published:** 2022-12-16

**Authors:** Nuthan Jagadeesh, Ambareesh Parameshwar, Hiranya Kumar, Vishwanath Shivalingappa

**Affiliations:** 1 Trauma and Orthopedics, Vydehi Institute of Medical Sciences and Research Centre, Bengaluru, IND; 2 Trauma and Orthopedics, Wrightington Wigan and Leigh National Health Service (NHS) Foundation Trust, Wigan, GBR

**Keywords:** blood transfusion in arthroplasty, navigation instrumentation, blood loss in total knee arthroplasty, hand-held navigation system, accelerometer-based navigation system

## Abstract

Introduction

Total Knee Arthroplasty (TKA) can be associated with significant peri- and post-operative blood loss necessitating blood transfusion. The blood loss may be relatively less when the accelerometer-based handheld navigation system (HHNS) is used, as there is neither a need for intramedullary breach nor additional pin insertions. The primary hypothesis was that HHNS instrumentation reduced perioperative blood loss when compared with conventional instrumentation, and to prove this, we compared the perioperative parameters like tourniquet time, hemoglobin loss, and estimated blood loss between patients undergoing total knee arthroplasty using conventional instrumentation with handheld navigation instrumentation.

Methods

This prospective comparative study involves 40 patients in the HHNS group and 40 patients in the conventional group based on the instrumentation used, respectively. Tourniquet was used in all the cases. Patient characteristics like age, sex, body mass index (BMI), American Society of Anaesthesiologists (ASA) grade, and Charlson Comorbidity Index (CCI) were recorded. The perioperative parameters like tourniquet time, the estimated blood loss, hemoglobin loss, blood transfusions, and the number of units transfused were recorded and compared between the groups.

Results

There was no significant difference in age, BMI, ASA grade, or CCI between the two groups. The tourniquet time was 83.7 ± 9.6 in the navigation and 73.9 ± 10.3 in the conventional group. The estimated Hb loss was lower at 2.5 ± 1.6 in the HHNS group compared to 3.0 ± 1.8 in the conventional group (p<0.001). Similarly, estimated blood loss was also lower at 830 ± 285ml for the HHNS group compared to 1088 ± 228 in the conventional group. Two patients in the navigation group had a total of four units transfused, whereas three patients in the conventional group had five units of blood transfusion.

Conclusions

The primary hypothesis that HHNS reduced perioperative blood loss was confirmed by the results of our study. We demonstrated that HHNS instrumentation significantly decreased the estimated blood loss, drain volume, and hemoglobin loss compared to conventional instrumentation with similar operating times. Though blood transfusions were seen in fewer patients, there was no significant reduction in blood transfusions by HHNS instrumentation.

## Introduction

Total Knee Arthroplasty (TKA) is common and increasingly routine surgery for the treatment of symptomatic knee osteoarthritis refractive to conservative treatment. The procedure can result in significant reliable outcomes, specifically improved quality of life, alleviating pain, restore function [1,2]. TKA is performed for primary osteoarthritis, but other potential indications include inflammatory arthritis, post-traumatic fractures, etc. There are various instrumentation systems adapted for TKA, which include conventional, navigation assisted, and robotic techniques. Compared to conventional techniques, navigation and robotic techniques are said to offer better alignment, performance, and survival rates [3,4].

TKA can be associated with significant peri- and post-operative blood loss necessitating blood transfusion [5]. A systematic review assessing allogeneic blood transfusion in total knee arthroplasty (TKA) found that it is required at variable rates in different series with a weighted average of 44 % (range 9% to 84 %) [6]. Perioperative optimization is essential to curtail the morbidity associated with blood loss and to reduce the burden of blood transfusion.

Blood transfusions are lifesaving; however, it is associated with risks like acute transfusion-related lung injury, the transmission of pathogens, etc., which can contribute to significant morbidity and mortality [7]. An essential goal of blood management is to eliminate the need for allogenic blood while preventing anemia. This minimizes transfusion risk, maximizes hemoglobin (Hb) status, and improves oxygen-carrying capacity, resulting in better recovery and long-term health.

The main source of bleeding is the breach in the femoral canal for the passage of the intramedullary rod in the conventional method. An accelerometer-based handheld navigation system (HHNS) is a palm-sized navigation device that helps to improve alignment [8-10]. The blood loss may be less in the HHNS as reference sensors are used to guide the component position instead of the intramedullary devices, but there is limited evidence regarding this in the literature. As the elderly population grows, TKA rates are on the rise, and people with a wide range of co-morbidities are more likely to need blood transfusions after undergoing surgery. Therefore, it is necessary to determine whether HHNS reduces postoperative blood loss, thereby reducing the need for blood transfusions.

The primary hypothesis was that HHNS instrumentation reduced perioperative blood loss when compared with conventional instrumentation, and to prove this, we compared the perioperative parameters like tourniquet time, hemoglobin loss, and blood loss between patients undergoing total knee arthroplasty using conventional instrumentation with handheld navigation instrumentation.

## Materials and methods

This prospective study involved 80 patients who underwent TKA between Jan 2019 to Jan 2020. Forty consecutive patients who underwent unilateral TKA using conventional instrumentation were allotted to the conventional group, and 40 consecutive patients who underwent unilateral TKA using HHNS instrumentation were allotted to the HHNS group. Informed consent was obtained from all the patients, and the study was approved by the Institutional ethical committee. Patients with bilateral knee replacement, surgeries warranting metal augments or extended stems, revision surgeries, patients who had femoral or tibial bone defects, fixed flexion deformities >10 degrees, and any active infection or major spine or hip abnormalities were excluded. In addition, patients with thrombosis, active thromboembolic disease, bleeding problems, or clotting disorders were excluded. Also excluded were patients who refused transfusions or were taking anticoagulants. Patient characteristics like age, sex, body mass index (BMI), American Society of Anaesthesiologists (ASA) grade, and Charlson Comorbidity Index were recorded.

All surgeries were performed by two senior authors who have been using HHNS for at least six months (> 30 surgeries) before the study period. All surgeries were performed under a tourniquet using a medial parapatellar approach. Tourniquet was inflated before the skin incision and was released after the closure for all the cases. 1g of tranexamic acid each was used both intravenously as well topically for all the patients. The drain was applied to all the cases and was removed 24hr after the surgery; the drain volume was documented before removal. Patients in the conventional group had TKA using conventional instrumentation with an intramedullary jig for femur cuts and an extramedullary jig for the tibial cut. In the conventional method, a hole was drilled in the femur for insertion of the intramedullary instrumentation, and at the end of the operation, the hole was not plugged with bone. Patients in the HHNS group underwent TKA using HHNS (Knee align 2) instrumentation using a surgical technique previously described [11]. All the patients had low molecular weight heparin for 14 days as thromboprophylaxis following surgery.

The preoperative variables recorded include age, sex, weight, height, BMI, and preoperative hemoglobin (Hb). Post-operative variables recorded include tourniquet time, hemoglobin 24hrs after surgery, drain volume, length of hospital stay, number of units transfused, and the number of patients needing a transfusion. The threshold for transfusion was 80g/l as per institutional protocol. Hb loss and estimated blood loss were calculated using the Hb balance method [12] using the following formulae

Hbloss = BV × (Hbi - Hbe) × 0.001 + Hbt

Estimated blood loss = 1000 × Hbloss / Hbi

where Hbloss(g): The loss volume of Hb; Hbi (g/L): The Hb value before surgery; Hbe (g/L): The Hb value after surgery; Hbt (g): The total volume of blood transfusion. A blood volume estimate (BV) (mL) is computed using Nadler's method, which considers a patient's weight, height, and gender [13]. The variables collected were compared between the groups.

Sample size calculation

It was expected that 70 patients (35 per group) would show a decrease in the primary outcome variable from 13.2 in the control/conventional group to 11.1 in the experimental group, with an expected standard deviation (SD) of 2.71 (5% significance level).

The calculation was based on the formula:

n = f(α/2, β) × 2 × σ2 / (μ1 − μ2)2

where μ1 and μ2 are the mean outcomes in the HHNS and CONV groups, respectively, and σ is the SD.

Statistical analysis

Statistical analysis was performed using IBM Corp. Released 2021. IBM SPSS Statistics for Windows, Version 28.0. Armonk, NY: IBM Corp. Categorical data were presented as numbers or percentages, and the continuous data were presented as mean +/_ standard deviation. The variables like demographics, laboratory results, drain volume, and the number of transfusions were performed using the Mann-Whitney U test for continuous and Chi-square test for categorical variables. A p-value of <0.05 was considered a statistically significant value.

## Results

Forty patients were considered in each group. The mean age of the patients in the study cohort was 64.6 ± 7.4 in the HHNS group and 66.8 ± 6.2 in the conventional group. Osteoarthritis was the major indication for the surgery, which accounted for 87.5% (n=70) cases. When ASA, CCI, and BMI are considered, there were no significant differences between the groups (p>0.05). The tourniquet time was 83.7 ± 9.6 in the navigation and 73.9 ± 10.3 in the conventional group (Tables 1, 2).

**Table 1:** Comparison of demographic variables, body mass index (BMI), American Society of Anaesthesiologists (ASA) grade, and Charlson Comorbidity Index (CCI) between two groups BMI: Body mass index, ASA: American Society of Anaesthesiologists grade, CCI: Charlson Comorbidity Index

Parameter		HHNS group	Conventional group	P value
Total number of cases		40	40	NA
Age (years ± SD)		64.6 ± 7.4	66.8 ± 6.2	0.733
Sex	Male	18	16	0.371
	Female	22	24	
Indication	Osteoarthritis	34	36	0.484
	Inflammatory arthritis	4	3	
	Other	2	1	
ASA	1	2	1	0.884
	2	7	9	
	3	29	30	
Charlson Comorbidity Index		2.7 ± 1.4	3.1 ± 1.8	0.581
BMI (kg/m2 ± SD)		29.4 ± 3.6	28.3 ± 4.7	0.869

**Table 2:** Comparison of perioperative parameters like tourniquet time, pre- and post-operative hemoglobin, estimated blood loss, drain volume, blood transfusion, length of hospital stay between handheld navigation group and conventional group.

Parameter	HHNS group	Conventional group	P value
Preoperative Hemoglobin	13.4 ± 1.2	13.1 ±1.4	0.786
Postoperative Hemoglobin (Day 1)	10.9 ± 1.8	10.1 ± 2.1	<0.001
Hemoglobin loss (Hbloss)	2.5 ± 1.6	3.0 ± 1.8	<0.001
Estimated blood loss(ml)	830 ± 285	1088 ± 228	<0.001
Drain volume (Day 1) (ml)	280 ± 84.2	336 ± 96.2	<0.001
No. of patients transfused	2	3	0.551
No. of units transfused	4	5	0.638
Tourniquet time	83.7 ± 9.6	73.9 ± 10.3	0.112
Hospital stays (days)	3.5 ± 2.4	3.8 ± 3.2	0.538

The average preoperative hemoglobin in the HHNS group and the conventional group was 13.4 ± 1.2 and 13.1 ±1.4, respectively. The post-operative hemoglobin was 10.9 ± 1.8 in the HHNS group and 10.1 ± 2.1 in the conventional group (p<0.001), which correlated with a drain volume of 280 ± 84.2 for the HHNS group and 336 ± 96.2 for the conventional group. The estimated Hb loss was lower at 2.5 ± 1.6 in the HHNS group compared to 3.0 ± 1.8 in the conventional group (p<0.001). Similarly, estimated blood loss was also lower at 830 ± 285ml for the HHNS group compared to 1088 ± 228ml in the conventional group. Two patients in the navigation group had a total of four units transfused, whereas three patients in the conventional group had five units of blood transfusion.

## Discussion

The newer instrumentation used for TKA, like computer navigation, robotic surgery, etc., is expected to give more accurate alignment of components leading to improved longevity of the implant. HHNS is an accelerometer-based navigation device that guides the surgeon for the more accurate distal femur and proximal tibial cuts avoiding component malalignment when compared to conventional instrumentation [11,14-16]. Furthermore, HHNS does not need to breach the intramedullary cavity in the femur, decreasing blood loss, but there is limited evidence in the literature suggesting this. We aimed to compare the perioperative characteristics, like estimated blood loss and blood transfusions, between HHNS and conventional instrumentation.

An analysis of 79 patients who underwent TKA by Xu et al. reported that the handheld navigation system increased operative time significantly compared with conventional instrumentation [17]. However, the prior experience of the surgeons in using these devices is not mentioned in the study. Further, the authors acknowledged that with experience, operating time would improve. Ikawa et al. also found that tourniquet time and femur resection time were higher in the accelerometer-based navigation system [18]. The antithrombin III and protein C pathways are activated during prolonged anoxia caused by longer tourniquet time [19]. As a result, further post-tourniquet bleeding may occur, negating the advantage of not opening the femoral canal for a navigated TKA [20]. The experience of the surgeon has been emphasized to reduce operating time. The operating time may not be comparable to a conventional system until 20-30 cases are done using a navigation system [21,22]. In our study, there was no difference in tourniquet time between the HHNS group and the conventional group. The senior author’s experience with the HHNS instrumentation may have made the operation quicker due to their familiarity with the instrumentation.

TKA may lead to considerable blood loss during the surgery. Several measures, like using a tourniquet, antifibrinolytic agents like tranexamic acid, and minimally invasive techniques, have all been tried to control blood loss. Gao et al., in their study involving 204 patients, reported that an accelerometer-based navigation system reduced hidden blood loss postoperatively, causing less decrease in the hemoglobin postoperatively [23]. The HHNS system does not require pin insertion like computer navigation, and it also causes less injury to the intramedullary canal because no intramedullary canal breach is necessary. The systemic review by Li et al. found that TKA using the HHNS system leads to reduced blood loss compared to the conventional technique. They attributed this to less damage femoral marrow cavity [24]. Conversely, some studies suggest that there is no difference in blood loss between navigation and conventional techniques [25,26]. In our study, both estimated blood loss and hemoglobin loss were lesser in the HHNS group when compared to the conventional group. Moreover, blood transfusion was seen in fewer patients in the HHNS group, but the difference was not significant. Furthermore, the drain volume was also significantly high in the conventional group. We attribute this again due to the lack of breach of the medullary cavity in this technique. As far as conventional techniques are concerned, a bone plug was not used in our study to block the medullary cavity. The systematic review by Yuenyongviwat et al. found that using a bone plug to block the intramedullary cavity significantly reduced blood loss and blood transfusions in conventional TKA [27]. It will be interesting to compare both HHNS and conventional techniques after using a bone plug to block the intramedullary canal in the conventional technique.

There are several limitations to our study. The study subjects were neither randomized nor blinded about the operating instrumentation. As a result of the HHNS instrumentation, an additional cost is incurred for the surgery, and the instrumentation is not evaluated for its cost-effectiveness. However, the study gives a reasonably accurate comparison of perioperative blood loss with well-matched study groups and is quite relevant to most operating surgeons in the world.

## Conclusions

The primary hypothesis that HHNS reduced perioperative blood loss was confirmed by the results of our study. We demonstrated that HHNS instrumentation significantly decreased the estimated blood loss, drain volume, and hemoglobin loss compared to conventional instrumentation with similar operating times. Though blood transfusions were seen in fewer patients, there was no significant reduction in blood transfusions by HHNS instrumentation.

## References

[1] Skou ST, Roos EM, Laursen MB, Rathleff MS, Arendt-Nielsen L, Simonsen O, Rasmussen S (2015). A randomized controlled trial of total knee replacement. N Engl J Med.

[2] Skou ST, Roos EM, Laursen MB, Rathleff MS, Arendt-Nielsen L, Rasmussen S, Simonsen O (2018). Total knee replacement and non-surgical treatment of knee osteoarthritis: 2-year outcome from two parallel randomized controlled trials. Osteoarthritis Cartilage.

[3] Fu Y, Wang M, Liu Y, Fu Q (2012). Alignment outcomes in navigated total knee arthroplasty: a meta-analysis. Knee Surg Sports Traumatol Arthrosc.

[4] Lee CY, Lin SJ, Kuo LT (2014). The benefits of computer-assisted total knee arthroplasty on coronal alignment with marked femoral bowing in Asian patients. J Orthop Surg Res.

[5] Noticewala MS, Nyce JD, Wang W, Geller JA, Macaulay W (2012). Predicting need for allogeneic transfusion after total knee arthroplasty. J Arthroplasty.

[6] Barr PJ, Donnelly M, Cardwell C, Alam SS, Morris K, Parker M, Bailie KE (2011). Drivers of transfusion decision making and quality of the evidence in orthopedic surgery: a systematic review of the literature. Transfus Med Rev.

[7] Goodnough LT, Shuck JM (1990). Risks, options, and informed consent for blood transfusion in elective surgery. Am J Surg.

[8] Nam D, Dy CJ, Cross MB, Kang MN, Mayman DJ (2012). Cadaveric results of an accelerometer based, extramedullary navigation system for the tibial resection in total knee arthroplasty. Knee.

[9] Iorio R, Mazza D, Drogo P (2015). Clinical and radiographic outcomes of an accelerometer-based system for the tibial resection in total knee arthroplasty. Int Orthop.

[10] Ueyama H, Minoda Y, Sugama R (2019). An accelerometer-based portable navigation system improved prosthetic alignment after total knee arthroplasty in 3D measurements. Knee Surg Sports Traumatol Arthrosc.

[11] Jagadeesh N, Kumar H, Sarparaju V, Shivalingappa V (2022). Comparative analysis of radiological evaluation and early functional outcomes of total knee arthroplasty using an accelerometer-based handheld navigation system and conventional instrumentation: A prospective study. Cureus.

[12] Gao FQ, Li ZJ, Zhang K, Sun W, Zhang H (2015). Four methods for calculating blood-loss after total knee arthroplasty. Chin Med J (Engl).

[13] Nadler SB, Hidalgo JH, Bloch T (1962). Prediction of blood volume in normal human adults. Surgery.

[14] Swamy AM, Malhotra R, Digge V, Manhas V, Gautam D, Srivastava DN (2022). Accelerometer-based portable navigation, a faster guide compared to computer-assisted navigation in bilateral total knee arthroplasty-a randomized controlled study. Knee Surg Sports Traumatol Arthrosc.

[15] Gao J, Hou Y, Li R, Ke Y, Li Z, Lin J (2021). The accelerometer-based navigation system demonstrated superior radiological outcomes in restoring mechanical alignment and component sagittal positioning in total knee arthroplasty. BMC Musculoskelet Disord.

[16] Shihab Z, Clayworth C, Nara N (2020). Handheld, accelerometer-based navigation versus conventional instrumentation in total knee arthroplasty: a meta-analysis. ANZ J Surg.

[17] Xu X, Liu P, Yuan Z (2019). Comparison of a novel handheld accelerometer-based navigation system and conventional instrument for performing distal femoral resection in total knee arthroplasty: a randomized controlled trial. Ann Transl Med.

[18] Ikawa T, Takemura S, Kim M, Takaoka K, Minoda Y, Kadoya Y (2017). Usefulness of an accelerometer-based portable navigation system in total knee arthroplasty. Bone Joint J.

[19] Murphy CG, Winter DC, Bouchier-Hayes DJ (2005). Tourniquet injuries: pathogenesis and modalities for attenuation. Acta Orthop Belg.

[20] Petaja J, Myllynen P, Myllyla G, Vahtera E (1987). Fibrinolysis after application of a pneumatic tourniquet. Acta Chir Scand.

[21] Jenny JY, Miehlke RK, Giurea A (2008). Learning curve in navigated total knee replacement. A multi-centre study comparing experienced and beginner centres. Knee.

[22] Smith BR, Deakin AH, Baines J, Picard F (2010). Computer navigated total knee arthroplasty: the learning curve. Comput Aided Surg.

[23] Gao X, Sun Y, Chen ZH, Dou TX, Liang QW, Li X (2019). Comparison of the accelerometer-based navigation system with conventional instruments for total knee arthroplasty: a propensity score-matched analysis. J Orthop Surg Res.

[24] Li J, Zhang Y, Gao X, Dou T, Li X (2022). Accelerometer-based navigation vs. conventional techniques for total knee arthroplasty (TKA): a systematic review and meta-analysis of randomized controlled trials. Arthroplasty.

[25] Thiengwittayaporn S, Junsee D (2009). Tanavalee A. A comparison of blood loss in minimally invasive surgery with and without electromagnetic computer navigation in total knee arthroplasty. J Med Assoc Thail.

[26] Singla A, Malhotra R, Kumar V, Lekha C, Karthikeyan G, Malik V (2015). A randomized controlled study to compare the total and hidden blood loss in computer-assisted surgery and conventional surgical technique of total knee replacement. Clin Orthop Surg.

[27] Yuenyongviwat V, Tuntarattanapong P, Iamthanaporn K, Hongnaparak T, Tangtrakulwanich B (2019). Intramedullary sealing with a bone plug in total knee arthroplasty to reduce blood loss: a meta-analysis of randomized controlled trials. J Orthop Surg Res.

